# Concomitant left atrial appendage exclusion during cardiac surgery: a single-center case series on technique profile, residual flow, and early safety

**DOI:** 10.1186/s13019-026-04017-9

**Published:** 2026-04-12

**Authors:** Masafumi Kudo, Hideki Tsubota, Yuki Akaguma, Masanori Honda, Hitoshi Okabayashi

**Affiliations:** Department of Cardiovascular Surgery, Mitsubishi Kyoto Hospital, 1 Katsuragosho-cho, Nishikyo-ku, Kyoto, 615-8087 Japan

**Keywords:** Left atrial appendage, Atrial fibrillation, Occlusion, Resection, Cardiac surgery, Residual flow, Suture techniques

## Abstract

**Background:**

Concomitant left atrial appendage exclusion is increasingly performed during cardiac surgery in patients with atrial fibrillation. However, real-world data on procedural variations, closure integrity, and early safety are limited.

**Methods:**

We retrospectively reviewed 86 patients with atrial fibrillation who underwent concomitant left atrial appendage exclusion during cardiac surgery at a single center between 2012 and 2025. Nineteen patients underwent left atrial appendage occlusion using suture-based techniques or a closure device, and 67 underwent left atrial appendage resection with closure using sutures or a stapler. Residual flow was assessed using intraoperative transesophageal echocardiography, and outcomes were evaluated during follow-up.

**Results:**

Patients in the occlusion group more often had longer atrial fibrillation duration, vitamin K antagonist use, and previous cardiac surgery. Mitral valve procedures were more frequently combined with occlusion, whereas maze ablation was more common in the resection group. Intraoperative residual flow was detected in three patients (16%) after occlusion and in none after resection. Both approaches showed favorable early safety, with no 30-day mortality and low reoperation rates due to bleeding. Differences in sinus rhythm and anticoagulation at three months were observed, but were likely influenced by concomitant maze procedures. Differences in long-term survival were also observed but should be interpreted cautiously because of baseline imbalances and unequal follow-up duration.

**Conclusions:**

In this single-center retrospective case series, suture-based occlusion was associated with residual flow in a subset of patients, whereas no residual flow was detected after resection using intraoperative echocardiography. Both approaches showed acceptable early safety. Larger multicenter studies with standardized follow-up imaging are needed to clarify closure durability and the long-term clinical outcomes.

## Background

The Left Atrial Appendage Occlusion Study III (LAAOS III) demonstrated that concomitant left atrial appendage (LAA) exclusion during cardiac surgery significantly reduced the incidence of stroke in patients with atrial fibrillation (AF) [1]. However, the choice of surgical technique for LAA exclusion remains heterogeneous in real-world practice, ranging from suture-based occlusion to anatomical resection. Although both approaches are widely adopted, data on their technical profiles, closure integrity, and perioperative safety in routine clinical settings remain limited [2, 3]. Therefore, further investigation of the characteristics and early outcomes of these techniques is warranted.

## Methods

### Study setting

We retrospectively reviewed patients with AF who underwent concomitant cardiac surgery with LAA occlusion (LAAO) or LAA resection (LAAR) at our institution. The study protocol was approved by the Institutional Review Board and adhered to the ethical principles of the Declaration of Helsinki. Given the retrospective design, individual informed consent was waived.

### Study population

The present study included consecutive patients who underwent concomitant cardiac surgery with LAAO or LAAR at our institution between November 2012 and March 2025.

### Procedures

The choice of LAA exclusion technique was determined by the operating surgeons. LAAO was performed using a single-layer purse-string suture, a two-layer continuous suture placed from within or outside the left atrium, or a closure device. LAAR involved excision of approximately 1 cm from the LAA root followed by closure with a two-layer continuous suture or a surgical stapler. Intraoperative transesophageal echocardiography (TEE) was routinely performed to evaluate the presence of residual flow into the LAA. Postoperative TEE to assess late residual flow or recanalization was not routinely performed. Postoperative outcomes were collected through outpatient visits and telephone follow-ups.

### Endpoints

The primary endpoints were the technical success of LAA exclusion, defined by the absence of residual flow on intraoperative TEE, and perioperative safety outcomes, including reoperation for bleeding and 30-day mortality. Secondary endpoints included maintenance of sinus rhythm at follow-up, continuation of anticoagulation therapy, and all-cause mortality during the observation period.

### Statistical analysis

All data were retrospectively obtained from the electronic medical records. Continuous variables are presented as mean ± standard deviation or median with interquartile range, and categorical variables are presented as counts and percentages. Comparisons between groups were performed using Fisher’s exact test for categorical variables and Student’s t-test or Mann–Whitney U test for continuous variables, as appropriate. Statistical analyses were performed using SPSS version 31 (IBM Corp., Armonk, NY, USA). A two-sided p-value < 0.05 was considered statistically significant.

## Results

A total of 86 patients with AF underwent concomitant left atrial appendage exclusion during cardiac surgery, including 19 patients in the LAAO group and 67 patients in the LAAR group.

### Pre-operative characteristics

Baseline characteristics are summarized in Table 1. The mean age was similar between groups (74 ± 8 years in both). The duration of AF was longer in the LAAO group than in the LAAR group (median 3 [7.5] vs. 0.5 [6.8] years, *p* = 0.023). Patients in the LAAR group received direct oral anticoagulants more frequently, whereas vitamin K antagonists were more common in the LAAO group (*p* < 0.001). A history of cardiac surgery was more frequent in the LAAO group (32% vs. 3%, *p* = 0.001). Other baseline characteristics, including comorbidities, CHADS₂-VASc score, and left atrial diameter, were comparable between the groups.

**Table 1:** Pre-operative patient characteristics

Variable	LAAO (*n* = 19)	LAAR (*n* = 67)	*p* value
Age, years, mean ± SD	74 ± 8	74 ± 8	0.82
Male, n (%)	14 (74%)	43 (64%)	0.59
Duration of AF, year, median [IQR]	3 [7.5]	0.5 [6.8]	0.023
Type of AF, n (%)			
Long-standing persistent	10 (53%)	21 (31%)	0.11
Persistent	1 (5%)	21 (31%)	0.034
Paroxysmal	8 (42%)	25 (37%)	0.79
Type of anticoagulant therapy, n (%)			
VKA	10 (53%)	10 (15%)	0.001
DOAC	3 (16%)	45 (67%)	< 0.001
CHADS₂-VASc score, mean ± SD	3.4 ± 1.5	3.3 ± 1.4	0.83
Medical history, n (%)			
HT	13 (68%)	45 (67%)	1.0
HL	6 (32%)	27 (40%)	0.6
DM	4 (21%)	9 (13%)	0.47
CKD (eGFR < 40 ml/min)	6 (32%)	39 (58%)	0.67
HD	0	4 (6%)	0.57
Smoking, former/current	10 (53%)	29 (43%)	0.6
Previous stroke	7 (37%)	11 (16%)	0.11
Previous cardiac surgery	6 (32%)	2 (3%)	0.001
Urgent surgery, n (%)	0	3 (4%)	1.0
LVEF < 50%, n (%)	4 (21%)	13 (19%)	1.0
LAD (mm), median [IQR]	49 [9]	44 [11]	0.04
JapanSCORE2, median [IQR]	3.2 [5.1]	3.2 [3.7]	0.35

*LAAO* left atrial appendage occlusion, *LAAR* left atrial appendage resection, *AF* atrial fibrillation, *VKA* vitamin K antagonist, *DOAC* direct oral anticoagulant, *HT* hypertension, *HL* hyperlipidemia, *DM* diabetes mellitus, *CKD* chronic kidney disease, *HD* hemodialysis, *LVEF* left-ventricular ejection fraction, *LAD* left atrial diameter, *SD* standard deviation, *IQR* interquartile range

Continuous variables are presented as mean ± standard deviation or median [interquartile range] depending on distribution and compared using Student’s t-test or Mann–Whitney U test as appropriate. Categorical variables are presented as counts (percentages) and compared using Fisher’s exact test

### Procedural characteristics

The procedural details are presented in Table 2. Cardiopulmonary bypass and cross-clamp times did not differ significantly between the groups. Mitral valve surgery was more frequently combined in the LAAO group (95% vs. 66%, *p* = 0.018), whereas concomitant left atrial maze ablation was more frequent in the LAAR group (39% vs. 5%, *p* = 0.005). The closure techniques for LAAO included single purse-string suturing (32%), double-layer suturing from within (32%) or outside the left atrium (32%), and one case with a closure device. LAAR was performed by cut-and-sew in 87% and by stapler in 13%.

**Table 2:** Procedural characteristics

Variable	LAAO (*n* = 19)	LAAR (*n* = 67)	*p* value
ECC time (min), median [IQR]	232 [102]	210 [85]	0.12
Cross-clamp time (min), median [IQR]	151 [75]	140 [76]	0.25
Surgical procedure, n (%)			
Isolated OPCAB	1 (5%)	6 (9%)	1.0
CABG	4 (21%)	10 (15%)	0.5
Mitral valve procedure	18 (95%)	44 (66%)	0.018
Aortic valve procedure	7 (37%)	13 (19%)	0.13
Tricuspid valve procedure	12 (63%)	32 (48%)	0.3
Any aortic procedure	0	5 (7%)	0.58
Other procedures	2 (11%)	9 (13%)	1.0
Concomitant surgical ablation, n (%)			
Full maze	1 (5%)	6 (9%)	1.0
Left atrial maze	1 (5%)	26 (39%)	0.005
PVI	2 (11%)	10 (15%)	1.0
Occlusion method, n (%)			
Single purse string suture from within LA	6 (32%)	NA	-
Double liner sutures from within LA	6 (32%)	NA	-
Double liner sutures from outside LA	6 (32%)	NA	-
Closure device	1 (5%)	NA	-
Resection method, n (%)			
Cut and sew	NA	58 (87%)	-
Stapler	NA	9 (13%)	-

*ECC* extracorporeal circulation, *OPCAB* off-pump coronary artery bypass, *CABG* coronary artery bypass grafting, *PVI* pulmonary vein isolation, *IQR* interquartile range, *NA* not applicable

Continuous variables are presented as mean ± standard deviation or median [interquartile range] depending on distribution and compared using Student’s t-test or Mann–Whitney U test as appropriate. Categorical variables are presented as counts (percentages) and compared using Fisher’s exact test

### Post-operative characteristics

**Fig. 1:**
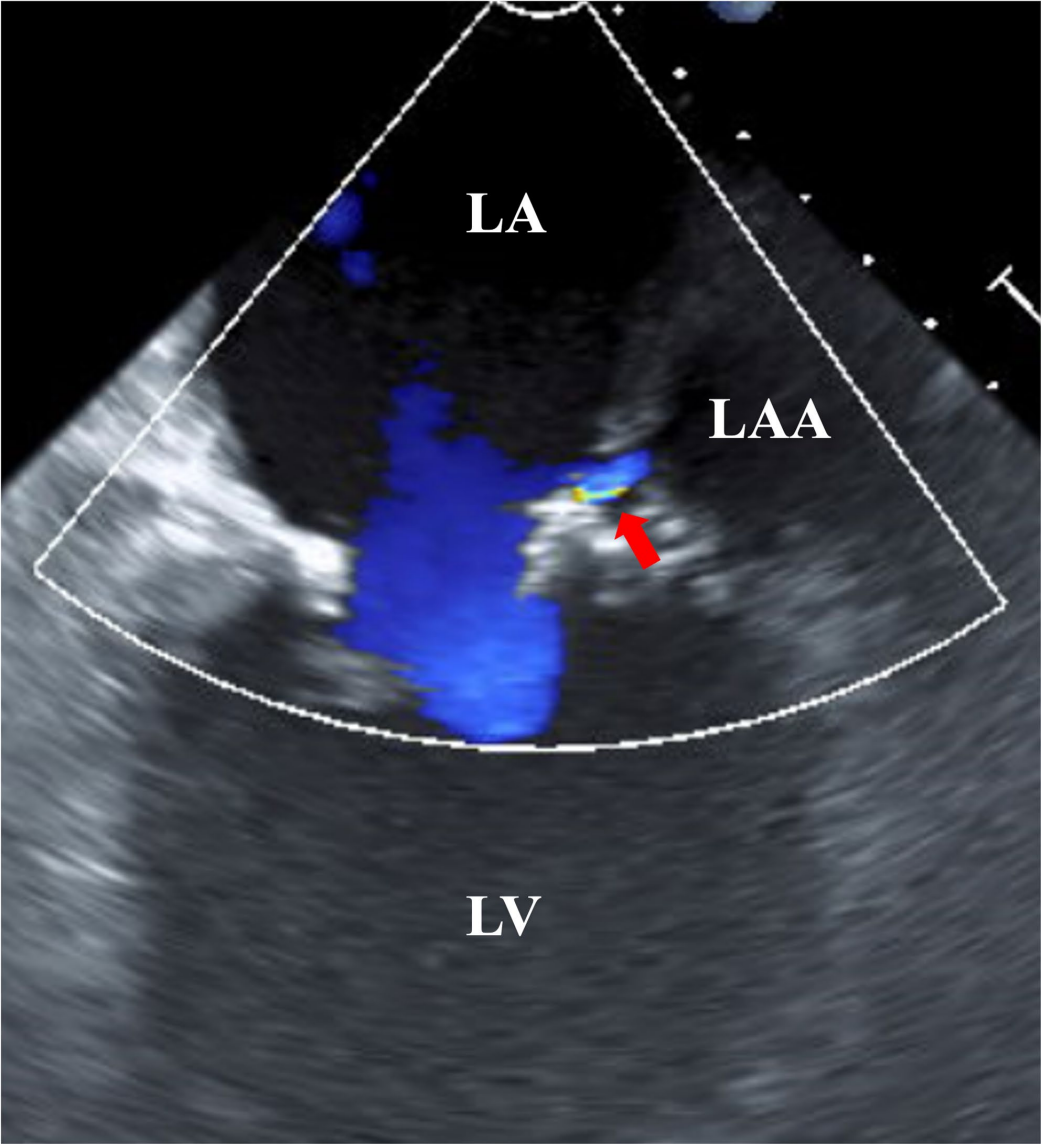
Intraoperative transesophageal echocardiography. TEE demonstrating residual flow (arrow) in a patient who underwent left atrial appendage occlusion with a single-layer purse-string suture. The arrow indicates persistent flow from the LAA to the LA cavity. *TEE* transesophageal echocardiography, *LA* left atrium, *LV* left ventricle, *LAA* left atrial appendage

Postoperative outcomes are presented in Table 3. The median follow-up duration was significantly longer in the LAAO group (81 months) than in the LAAR group (30 months, *p* = 0.004). Residual flow was detected intraoperatively in three patients (16%) in the LAAO group, including two cases after a two-layer continuous suture placed from outside the left atrium and one case after a single-layer purse-string suture (Fig. 1). No residual flow was observed after two-layer continuous suturing from the inside of the atrium or device closure; however, the number of patients undergoing these techniques was limited. Residual flow was not detected in the LAAR group (*p* = 0.009). Reoperation for bleeding occurred in two patients (11%) in the LAAO group and one patient (1.5%) in the LAAR group (*p* = 0.12). No 30-day mortality was observed in either group. During follow-up, overall mortality was higher in the LAAO group (32% vs. 10%, *p* = 0.034). At 3 months, sinus rhythm was maintained more often in the LAAR group (64% vs. 37%, *p* = 0.04), and the continuation of anticoagulation therapy was less frequent in the LAAR group (39% vs. 68%, *p* = 0.035). The rates of postoperative stroke, cerebral hemorrhage, and new pacemaker implantation were low and not significantly different between the groups. Given the substantial differences in follow-up duration and baseline characteristics between the groups, these between-group comparisons should be interpreted descriptively.

**Table 3:** Post-operative outcomes

Variable	LAAO (*n* = 19)	LAAR (*n* = 67)	*p* value
Follow-up (mo), median [IQR]	81 [96]	30 [37]	0.004
Residual flow into the LAA, n (%)	3 (16%)	0	0.009
Single purse string suture from within LA	1/6	-	-
Double liner sutures from within LA	0/6	-	-
Double liner sutures from outside LA	1/6	-	-
Closure device	0/1	-	-
Complication, n (%)			
Stroke	1 (5.3%)	1 (1.5%)	0.4
Cerebral hemorrhage	1 (5.3%)	0	0.22
Reoperation for bleeding	2 (11%)	1 (1.5%)	0.12
Death within 30 days	0	0	-
Death from any cause	6 (32%)	7 (10%)	0.034
Hospital stay (days), median [IQR]	20 [8]	18 [73]	0.19
Sinus rhythm at discharge, n (%)	8 (42%)	42 (63%)	0.12
Sinus rhythm at 3 months, n (%)	7 (37%)	43 (64%)	0.04
Anticoagulant at discharge, n (%)	19 (100%)	65 (82%)	1.0
Anticoagulant at 3 months, n (%)	13 (68%)	26 (39%)	0.035
New pacemaker implantation, n (%)	3 (16%)	6 (9%)	0.41

*LAAO* left atrial appendage occlusion, *LAAR* left atrial appendage resection, *IQR* interquartile range, *LAA* left atrial appendage

Continuous variables are presented as mean ± standard deviation or median [interquartile range] depending on distribution and compared using Student’s t-test or Mann–Whitney U test as appropriate. Categorical variables are presented as counts (percentages) and compared using Fisher’s exact test

## Discussion

The present study describes our institutional experience with concomitant LAA exclusion during cardiac surgery in patients with AF. In this retrospective single-center cohort study of 86 patients (19 LAAO and 67 LAAR), residual flow was observed intraoperatively in 16% of patients undergoing LAAO. Both procedures were performed with favorable perioperative safety, with no 30-day mortality and low rates of reoperation for bleeding observed. At three months, sinus rhythm was more frequently maintained and anticoagulation therapy was less often continued in the LAAR group; however, these differences were likely influenced by concomitant maze procedures. Overall survival was lower in the LAAO group, but this observation should be interpreted with caution because of baseline imbalances and substantially different follow-up durations between groups. Accordingly, the present findings are descriptive and hypothesis-generating, rather than definitive comparative evidence.

While the efficacy of LAA exclusion in reducing stroke risk has been demonstrated in large randomized trials such as LAAOS III, the optimal surgical technique in routine practice remains uncertain [1, 4–6]. Our findings provide real-world insights into the procedural characteristics, intraoperative closure integrity, and early outcomes of two commonly employed approaches: LAA occlusion and LAA resection. In our cohort, LAAO consisted of several suture-based techniques and one device-based closure technique. Residual flow was detected in a subset of patients undergoing LAAO, consistent with previous reports suggesting that suture-based techniques may be associated with incomplete closure [7, 8, 9]. Intraoperative TEE identified residual flow, particularly after a two-layer continuous suture was placed from outside the left atrium or a single-layer purse-string suture. In contrast, no residual flow was observed after two-layer continuous suturing from inside the atrium or after device closure; however, the number of patients undergoing these techniques was limited. These observations suggest that technical approaches may influence intraoperative closure integrity and highlight the importance of careful intraoperative assessment using TEE. Importantly, although none of the patients with intraoperative residual flow experienced stroke during the observation period, incomplete closure of the LAA has been associated with thrombus formation and increased embolic risk in prior studies [2, 3, 10]. However, our evaluation was limited to intraoperative TEE, and late residual communication or recanalization after surgery was not routinely assessed using postoperative TEE. Therefore, the long-term clinical relevance of residual flow in this cohort remains uncertain.

LAAR was associated with complete exclusion without residual flow on intraoperative TEE, reflecting anatomical certainty of excision. From a practical standpoint, these findings suggest that LAAO may offer technical simplicity, whereas LAAR may provide more consistent exclusion at the expense of additional surgical manipulation and a potential risk of bleeding than LAAO. Nevertheless, due to baseline imbalances, technique heterogeneity within groups, and limited event numbers, the present study does not allow conclusions regarding the superiority of one approach over the other.

Perioperative safety outcomes were favorable in both groups, with no 30-day mortality and low rates of major complications. Reoperation for bleeding occurred infrequently, and the duration of hospital stay was comparable between the groups. These results support the overall feasibility and safety of concomitant LAA exclusion during cardiac surgery.

Secondary observations suggested differences in rhythm status and anticoagulant management. Patients in the LAAR group were more often in sinus rhythm at three months and were less likely to continue anticoagulation therapy. However, these findings should be interpreted cautiously because concomitant maze procedures were more frequently performed in the LAAR group, which likely affected rhythm outcomes and subsequent anticoagulant decisions. Similarly, the observed differences in long-term survival are likely confounded by baseline patient characteristics and unequal follow-up duration. Although the EAST-AFNET 4 trial demonstrated that early rhythm control reduces cardiovascular outcomes including stroke in patients with AF, our study did not directly assess rhythm-control strategies, and the findings should not be extrapolated beyond this descriptive context [11]. Future prospective multicenter studies with standardized surgical protocols, rhythm management strategies, and postoperative imaging follow-up are needed to clarify the durability of LAA closure and its association with clinical outcomes, including stroke prevention and anticoagulation strategy.

### Limitations

This study had several limitations. First, this was a retrospective, single-center series with a relatively small sample size and imbalance between the two groups, reflecting non-randomized real-world surgical decision-making and a potential selection bias. Second, the technique heterogeneity within each group limits technique-specific comparisons and precludes conclusions regarding superiority. Third, the completeness of LAA exclusion was assessed primarily by intraoperative TEE, and standardized postoperative TEE to evaluate late residual flow or recanalization was not routinely performed. In addition, although guideline-based definitions of complete LAA exclusion include both the absence of residual flow and a stump length of < 1 cm, stump length was not systematically measured or documented in this cohort, particularly in the LAAO group. Fourth, differences in the follow-up duration and concomitant maze procedures between the groups further limited the comparability of the outcomes. Therefore, our findings should be interpreted as descriptive and hypothesis-generating, rather than as definitive comparative evidence. Despite these limitations, our report provides a practical overview of real-world technique profiles, highlights intraoperative residual flow as a relevant technical consideration after LAAO, and supports the feasibility and early safety of concomitant LAA exclusion during cardiac surgery.

## Conclusions

This single-center retrospective case series provides a descriptive overview of real-world concomitant LAA exclusion during cardiac surgery in patients with AF. Both LAAO and LAAR were performed with acceptable perioperative safety and no 30-day mortality. Intraoperative residual flow was observed in a subset of patients undergoing suture-based LAAO, whereas no residual flow was detected after LAAR on intraoperative TEE. Differences in rhythm status and anticoagulation use were observed between the groups; however these findings should be interpreted cautiously, given the baseline imbalances and the higher rate of concomitant maze procedures in the LAAR group. Overall, our findings highlight the importance of intraoperative assessment of closure integrity and support the need for larger multicenter studies with standardized follow-up imaging to clarify the durability and long-term clinical implications of the different surgical LAA exclusion strategies.

## Data Availability

The datasets generated and analyzed during the current study are not publicly available due to patient privacy but are available from the corresponding author on reasonable request.

## Abbreviations

LAAOS III: Left Atrial Appendage Occlusion Study III
LAA: Left Arial Appendage
AF: Atrial fibrillation
LAAO: Left Atrial Appendage Occlusion
LAAR: Left Atrial Appendage Resection
TEE: Transesophageal Echocardiography
VKA: Vitamin K Antagonist
DOAC: Direct Oral Anticoagulant
HT: Hypertension
HL: Hyperlipidemia
DM: Diabetes Mellitus
CKD: Chronic Kidney Disease
HD: Hemodialysis
LVEF: Left-Ventricular Ejection Fraction
LAD: Left Atrial Diameter
SD: Standard Deviation
IQR: Interquartile Range
ECC: Extracorporeal Circulation
OPCAB: Off-Pump Coronary Artery Bypass
CABG: Coronary Artery Bypass Grafting
PVI: Pulmonary Vein Isolation
LV: Left Ventricle
